# Correlation Between Maternal Age and Cytokine (IL-6 and TGF-Beta) Levels in Colostrum

**DOI:** 10.7759/cureus.39146

**Published:** 2023-05-17

**Authors:** Shivani, Sandeep Bhattacharya, Wahid Ali, Archana Ghildiyal

**Affiliations:** 1 Physiology, King George's Medical University, Lucknow, IND; 2 Pathology, King George's Medical University, Lucknow, IND

**Keywords:** cytokine, tumor growth factor-beta (tgf-b), interleukin-6 (il-6), colostrum, advancing maternal age

## Abstract

Background: Cytokines are small proteins that play an important role in cell signaling, particularly in inflammatory pathways. There are both pro- as well as anti-inflammatory cytokines that regulate this pathway and modulate the immune responses. Advancing maternal age is associated with systemic inflammation. The present study intends to evaluate the effect of advancing maternal age on cytokine (IL-6 and TGF-β) levels in colostrum, the first breast milk secreted by mothers.

Methodology: A total of 77 term deliveries were enrolled in the study. Colostrum specimens were collected and evaluated for cytokine IL-6 and TGF-β levels. Colostrum IL-6 and TGF-β levels were correlated with maternal age and were assessed using the Spearman rank correlation coefficient. Multivariate analysis was done using a linear regression model comprising age, parity, and mode of delivery.

Results: Mean colostrum IL-6 and TGF-β levels were 113.3±73.1 pg/ml and 20.9±23.6 pg/ml, respectively. No significant correlation between maternal age and colostrum IL-6 levels was observed (r=0.137; p=0.314). However, there was a significant positive correlation between maternal age and colostrum TGF-β levels (r=0.452; p<0.001).

Conclusions: The findings of the study show a significant association between maternal age and colostrum TGF-β levels. The impact of colostrum cytokine levels on neonatal growth and development in context with advancing maternal age needs to be evaluated.

## Introduction

Colostrum is the first breast milk that is “produced for the first few days after giving birth and is unique in its composition of essential nutrients, immune factors, and oligosaccharides that benefit the newborn” [1,2]. Compared to mature breast milk, colostrum is characterized by a higher proportion of protein and a lower proportion of carbohydrates and fats. It also has immunoglobulin A (IgA) which is helpful in protecting infants from infections. Colostrum also establishes a normal gut microbiome in the infant [3].

It has always been of interest whether colostrum characteristics are affected by maternal nutritional and health status. Relationship between maternal circulating vitamin status and colostrum vitamin composition [4], lactoferrin levels with maternal nutritional status [5], and micronutrient levels [6], etc. have been explored in various previous studies, indicating that maternal health state is reflected in colostrum. Colostrum properties have been shown to be affected by a host of factors including maternal age, ethnicity, geography, diet, BMI, parity, gestational age at delivery, season, and pregnancy complications [7-13]. Among these, maternal age is one of the most important factors affecting colostrum characteristics [14-16]. In the present study, we assessed cytokine IL-6 and TGF-b levels in colostrum and evaluated their association with maternal age, parity, and mode of delivery.

## Materials and methods

Study design and participants

This is a cross-sectional study that assessed colostrum IL-6 and TGF-β levels in women who recently attained motherhood. A total of 77 new lactating mothers were enrolled in the study within 48 hours of giving birth at the Department of Obstetrics and Gynaecology, King George’s Medical University, Lucknow, India.

Inclusion and exclusion criteria

The following inclusion criteria were applied: healthy lactating mothers who delivered within 48 hours, aged 18 years or above. Participants were excluded based on: chronic illness, diabetes mellitus/history of gestational diabetes, hypertension/history of pregnancy-induced hypertension (PIH) or preeclampsia, endocrine disorders, being currently on medication, twin or multifetal deliveries.

Ethical considerations

This study was carried out as per the guidelines of the Helsinki Declaration. Participation in the study was entirely voluntary, and the participants were included in the study after obtaining informed consent from patients following a detailed description of the study procedure and possible benefits and risks involved. The project was approved by the Institutional Ethics Committee of King George's Medical University (approval no. 995/Ethics/2021) before the enrolment of participants.

Collection of demographic and obstetric data

After obtaining ethical approval for the study, women fulfilling the inclusion criteria were asked to participate in it. A thorough clinical and medical history was obtained in order to ascertain that they did not fall under the exclusion criteria. Only lactating mothers who fulfilled the selection criteria were recruited for the study. Following enrolment, their age, parity, and mode of delivery were noted.

Collection of colostrum

The colostrum was manually expressed for collection from 8 a.m. to 10 a.m. and in the interval between two feedings, at 24 and 48 hours after delivery. From each patient, a total of 2 ml to 3 ml of colostrum was collected into sterile plastic tubes, which were immediately transported to the laboratory in an ice box.

Evaluation of cytokine levels

Samples were centrifuged at 13,000 rpm for 15 minutes. The fat layer was discarded, and the aqueous component was stored at -70°C. Thawing of the specimens was done at 4°C. For further analysis, a total of 2 ml of the specimen was placed in a centrifugation machine and centrifuged using 160 g of relative centrifugal force.

Centrifugation was done at a temperature of 4°C for a duration of 10 minutes. The centrifuged contents comprised three layers: the bottom layer had a cell pellet, the middle layer comprised aqueous contents, and the topmost layer had a deposition of fats. The topmost layer with the fats was discarded, and then the middle aqueous supernatant was separated for the purpose of estimating cytokine levels. Cytokine assessment was done using enzyme-linked immunosorbent assay (ELISA) kits for quantified estimation of IL-6 and TGF-β. The assays were performed as per the instructions of the manufacturer [15].

Statistics

The data obtained were entered in Microsoft Excel 2013 software (Microsoft Corp., Redmond, WA, USA). Statistical analysis was performed with the help of SPSS Statistics (IBM Inc., Armonk, NY, USA). Categorical data were represented as numbers (frequency) and percentages (proportions), and continuous data were represented as mean±standard deviation (SD) and median (IQR). Bivariate correlation was assessed using Spearman’s rho coefficient. Linear regression was performed to assess multivariate associations.

## Results

The age of mothers ranged from 20 to 43 years. The mean age of mothers was 31.3±6.2 years (median 34 years; IQR 25 to 36.38 years). There were 28 (36.4%) women aged >35 years. The maximum number of women were in para 1 (44.6%) followed by para 2 (30.4%), para 3 (19.6%), and para 4 (5.4%), respectively. The majority of women delivered vaginally (72.7%). There were 21 (27.3%) cesarean deliveries. All the cesarean deliveries took place in women aged <35 years. Colostrum IL-6 levels ranged from 2.52 to 297.05 pg/ml with a mean of 113.3±7.31 pg/ml. The median IL-6 level was 113.2 pg/ml with an IQR from 51.8 to 184.4 pg/ml. Colostrum TGF-β levels ranged from 6.8 to 128.66 pg/ml with a mean of 20.9±23.6 pg/ml. The median colostrum level was 14.4 pg/ml with an IQR from 10.8 to 19.4 pg/ml (Table 1).

**Table 1:** Demographic profile and characteristics of the study population *Women who delivered vaginally were aged <35 years

Characteristics	Statistic
Maternal age±SD in years | Median age (IQR in years)	31.3±6.2 (20-43) | 34 (26-36.38)
Number of women aged >35 years	28 (36.4%)
Parity	
P1	25 (44.6%)
P2	17 (30.4%)
P3	11 (19.6%)
P4	3 (5.4%)
Mode of delivery	
Vaginal	56 (72.7%)
Cesarean	21 (27.3%)*
Mean Colostrum IL-6 level±SD in pg/ml | Median Colostrum IL-6 level (IQR in pg/ml)	113.3±73.1 (2.52-297.05) | 113.2 (51.8-184.4)
Mean Colostrum TGF-β level±SD in pg/ml | Median Colostrum TGF-beta level (IQR in pg/ml)	20.9±23.6 (6.8-128.66) | 14.4 (10.8-19.4)

**Figure 1:**
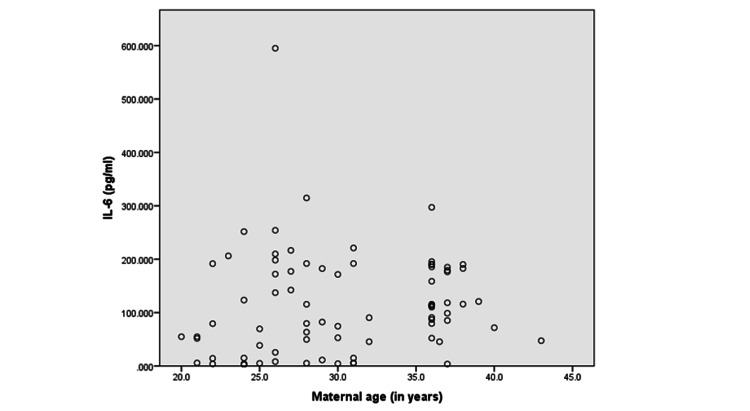
Correlation between maternal age and IL-6 levels (rho=0.137, p=0.314)

**Figure 2:**
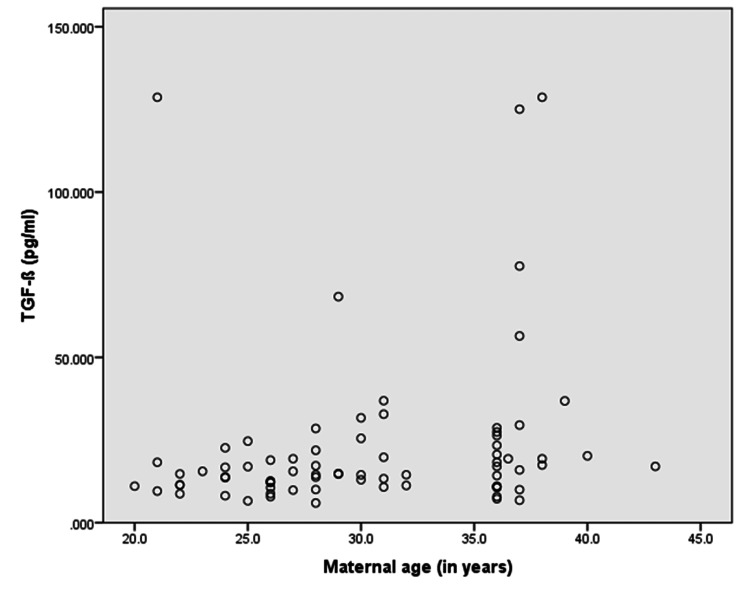
Correlation between maternal age and TGF-β levels (rho=0.452, p<0.001)

On linear regression, where IL-6 and TGF-β levels were considered dependent on the independent variables of age, parity, and mode of delivery, none of the independent factors were found to be significantly associated with IL-6 levels (p>0.05). However, for TGF-β levels, only age emerged as a significant predictor (p=0.026). The correlation of maternal age with IL-6 resulted in a weak positive but non-significant correlation (r=0.137; p=0.314) whereas the correlation between maternal age and TGF-β level resulted in a positive significant correlation (r=0.451; p<0.001) (Table 2; Figures 1-2).

**Table 2:** Linear regression for the association of colostrum IL-6 and TGF-β levels with maternal characteristics SE: Standard error

Variable	IL-6				TGF-β		
	b±SE	t-value	p-value	b±SE		t-value	p-value
Constant	55.68±64.30	0.866	0.389	-8.90±15.88		-0.560	0.577
Age	2.80±2.17	1.292	0.200	1.22±0.54		2.279	0.026
Parity	-16.13±14.23	-1.133	0.261	-4.49±3.52		-1.276	0.206
Caesarean delivery (0=No, 1=Yes)	11.10±26.00	0.427	0.679	8.77±6.42		1.366	0.176
	r^2^=0.030			r^2^=0.081			

## Discussion

Advancing age is considered a high risk for childbearing and is known to result in both physical and physiological complications. It is also associated with complications during pregnancy, with an increased frequency of adverse antenatal and postnatal events [17]. Even after the culmination of pregnancy, it has a consequent impact on both maternal and fetal well-being. The impact of advanced maternal age is not limited to the delivery of a baby but also affects childrearing and feeding patterns. Mothers of advanced age are known to have low compliance with exclusive breastfeeding as they often hold a perception of low milk supply [18-20]. In the present study, we evaluated the colostrum cytokine levels IL-6 and TGF-β in context with advancing maternal age, parity, and mode of delivery and found a significant positive correlation of TGF-β levels with maternal age. However, no such correlation was seen between IL-6 levels and maternal age. Compared to the present study, Ferrari et al. found a significant association of IL-6 levels with advancing age, however, they did not find this relationship to be linear and found the IL-6 levels to be significantly higher in advanced-age mothers (median age 37 years) as compared to adolescent mothers with a median age of 20 years [15]. In the present study, we did not have any mothers aged <20 years, and hence we are not in a position to comment on this association. Interestingly, in Ferrari et al.'s study, none of the other cytokines evaluated (IL-1b, IL-8, and TGF-α) showed a significant association with age [15]. One of the reasons for the difference in the nature of associations seen in the present study and theirs could be the time of evaluation.

In the present study, we took colostrum samples within 48 hours of delivery for assessment, as compared to 48 to 72 hours post-delivery for assessment in the study by Ferrari et al. [15]. It must be understood that TGF-β, a pro-inflammatory cytokine, is activated earlier compared to IL-6 which is an anti-inflammatory cytokine.

Advanced maternal age results in pronounced oxidative stress. The study by Gila-Diaz et al. found a significant association between maternal age and breast milk oxidative stress markers during the first month of lactation [21]. We also assume the increased colostrum inflammatory cytokine levels in mothers with advanced age to be reflective of this increased oxidative stress manifested in terms of an increase in inflammatory biomarker levels. However, keeping in view the low-risk profile of mothers in the present study, there could be relatively lower stress levels.

Factors like cesarean delivery [15], ethnicity, geography, diet, BMI, parity, gestational age at delivery, season, and pregnancy complications [7-13] have been shown to affect breastmilk composition. However, in the present study, owing to the selection criteria, except for parity, all the other factors were well controlled, and hence their impact could not be assessed. As far as parity is concerned, in the multivariate assessment, we found that it did not confound the TGF-β levels, and only advanced age emerged as a significant predictor of TGF-β levels. As far as the impact of parity on breast milk composition is concerned, it has primarily been reported in context with differences between primipara and higher parity and for fat and carbohydrate content in the breastmilk [22,23]; there are no studies evaluating this impact in terms of cytokine levels.

Although advanced age was found to be significantly associated with TGF-β levels, we feel that this problem should not be viewed in a univariate context and that the role of other possible factors such as diet, maternal BMI, pregnancy and health complications, term of delivery, mode of delivery, and other maternal and neonatal factors should also be taken into account.

The findings of the present study are thought-provoking; however, being preliminary in nature, they require further corroboration. Especially as such assessment of inflammatory biomarkers like IL-6 and TGF-β in the colostrum still remains unstandardized. There is no normalized or standard data available; hence, it is difficult to categorize whether they have any clinical impact or not. The findings of the present study indicate the need to standardize colostrum inflammatory cytokine levels with the help of a larger pool of data. The present study was limited by a small sample size and had highly selective inclusion criteria, which ruled out the possibility of evaluating the role of other factors that may influence the level of colostrum biomarkers.

## Conclusions

The present study was a pilot study exploring the association of advanced maternal age with colostrum composition in terms of cytokine levels. Despite its smaller sample size, the study was able to find that advancing age tends to affect the cytokine levels in colostrum. However, the mode of delivery did not seem to affect the colostrum cytokine levels. The findings of the study show that colostrum cytokine levels tend to be affected by advancing age. Whether it has an impact on the further growth and development of newborns remains to be examined. Further studies on a larger sample size with the inclusion of other variables of interest are recommended. Subsequent studies to evaluate the impact of colostrum cytokine levels on an infant's growth, development, and immunity are also recommended.
